# The MADS Box Genes *ABS*, *SHP1*, and *SHP2* Are Essential for the Coordination of Cell Divisions in Ovule and Seed Coat Development and for Endosperm Formation in *Arabidopsis thaliana*

**DOI:** 10.1371/journal.pone.0165075

**Published:** 2016-10-24

**Authors:** Katrin Ehlers, Amey S. Bhide, Dawit G. Tekleyohans, Benjamin Wittkop, Rod J. Snowdon, Annette Becker

**Affiliations:** 1 Justus Liebig University, Institute of Botany, Heinrich-Buff-Ring 38, D-35392, Gießen, Germany; 2 Justus Liebig University, Department of Plant Breeding, Heinrich-Buff-Ring 26-32, D 35392, Gießen, Germany; Ecole Normale Superieure, FRANCE

## Abstract

Seed formation is a pivotal process in plant reproduction and dispersal. It begins with megagametophyte development in the ovule, followed by fertilization and subsequently coordinated development of embryo, endosperm, and maternal seed coat. Two closely related MADS-box genes, *SHATTERPROOF 1* and *2* (*SHP1* and *SHP2*) are involved in specifying ovule integument identity in *Arabidopsis thaliana*. The MADS box gene *ARABIDOPSIS BSISTER* (*ABS* or *TT16*) is required, together with *SEEDSTICK* (*STK*) for the formation of endothelium, part of the seed coat and innermost tissue layer formed by the maternal plant. Little is known about the genetic interaction of *SHP1* and *SHP2* with *ABS* and the coordination of endosperm and seed coat development. In this work, mutant and expression analysis shed light on this aspect of concerted development. Triple *tt16 shp1 shp2* mutants produce malformed seedlings, seed coat formation defects, fewer seeds, and mucilage reduction. While *shp1 shp2* mutants fail to coordinate the timely development of ovules, *tt16* mutants show less peripheral endosperm after fertilization. Failure in coordinated division of the innermost integument layer in early ovule stages leads to inner seed coat defects in *tt16* and *tt16 shp1 shp2* triple mutant seeds. An antagonistic action of *ABS* and *SHP1/SHP2* is observed in inner seed coat layer formation. Expression analysis also indicates that *ABS* represses *SHP1*, *SHP2*, and *FRUITFUL* expression. Our work shows that the evolutionary conserved B_sister_ genes are required not only for endothelium but also for endosperm development and genetically interact with *SHP1* and *SHP2* in a partially antagonistic manner.

## Introduction

For seed setting, most angiosperms go through a double fertilization process, whereby two sperm cells released from a pollen tube fuse with the egg cell and the central cell within the female gametophyte. These later develop into a diploid embryo and the triploid endosperm constituting the seed which is surrounded by the seed coat originating from the maternal integuments of the ovule [1, 2, 3].

The endosperm is an essential part of the seed since it provides nutrients to the developing embryo during seed development [4]. In addition, endosperm and its associated aleurone layers are important for germination and subsequent seedling development [5, 6]. In *Arabidopsis thaliana*, the development of endosperm follows the nuclear type pattern whereby a repeated free nuclear division, without cytokinesis, is followed by a cellularization event [3]. Several studies indicated that proper endosperm development, nuclear division and cellularization are essential for the success of viable seed setting [7]. One of the factors that influence embryo and endosperm development is the maternal tissue, the integuments, whose communication with the endosperm is essential for seed development [8, 9, 10, 11]. Several genes, including MADS-box transcription factor encoding genes, have been implicated in the ovule integument and seed endosperm development [12, 13, 14, 15, 16, 17, 7, 18].

The MADS-box gene family is one of the most thoroughly investigated gene families. Members of this gene family encode proteins that are mainly characterized by their highly conserved DNA-binding domain of 58 amino acids known as the MADS-domain that specifically binds to CC(A/T)_6_GG motifs found in the regulatory regions of their target genes [19, 20, 21, 22, 23, 24].

The B_sister_ MADS box gene clade was named based on their close phylogenetic relationship to the B-class genes and on the fact that members of this clade are mainly expressed in female rather than in male reproductive organs [25]. Members of the B_sister_ gene clade are present in all angiosperms and gymnosperms investigated so far and they are reported to have a highly conserved ovular expression [25, 26].

The *ARABIDOPSIS* B_SISTER_ gene (*ABS*) was the first B_sister_ gene to be functionally characterized and the mutant allele *tt16-1* (*transparent testa 16–1*) defective in *ABS* function has been analyzed most extensively [27, 28]. *ABS* expression is restricted to the ovule, in the cells of which the protein is localized in the nucleus [29, 18]. *tt16-1* ovules show thinner inner integument cell layers and develop into a straw-colored seed, except in their chalaza-micropyle area, due to the inactivation of *BANYULS (BAN)*, a key regulator of proanthocyanidin (PA) biosynthesis [27, 28].

*ABS* orthologs (*BnTT161-4*) in *Brassica napus* (rapeseed canola) also regulate agronomically important traits such as seed oil content and fatty acid composition. *B*. *napus ABS* ortholog RNAi lines have seeds with flattened and wrinkled structure containing a defective embryo or completely lacking an embryo and altered fatty acid composition [30, 31]. Similar to eudicots, monocot B_sister_ genes are also reported to be involved in seed development [32, 33]. *OsMADS29*, one of the three B_sister_ homologs in rice (*Oryza sativa*), regulates the expression of genes that are essential for initiation of programmed cell death (PCD) in the nucellar region of developing seeds. Expressed mainly in the ovule, down-regulation of *OsMADS29* resulted in shrunken seeds with poor grain-filling rate [32, 33]. Another *OsMADS29* loss-of-function mutant allele, *fst (female sterile)* shows a phenotype including complete female sterility due to an absence of embryo and endosperm development [34].

Protein interaction analyses indicated that ABS forms heterodimers and tetramers with several MADS proteins, including the D-class floral homeotic proteins: SEEDSTICK (STK), SHATTERPROOF 1 and 2 (SHP1, SHP2) mediated by SEPALLATA 3 (SEP3) [35]. *ABS* and *STK* function redundantly to regulate the ovule inner integument formation; *abs stk* lacks the entire inner integument layer and, subsequently, the endothelium, resulting in extremely poor seed setting [18].

*SHP1* and *SHP2* are two closely related and highly redundant MADS-box genes involved in several aspects of reproductive development. They are best known for their promotion of lignification in the dehiscence zone, hence causing pod shattering at maturity. While single *shp* mutant fruits resemble those of the wild type, *shp1 shp2* double mutant fruits fail to dehisce, and plants that over-express the two genes show premature fruit opening at the valve margins [36]. Furthermore, *SHP1* and *SHP2* are required for ovule identity, together with *STK* and the class C floral homeotic gene *AGAMOUS* (*AG*). However, it remains unknown if the *SHP1* and *SHP2* carry non-redundant functions in seed development. Using SEP3 as a bridging protein, all combinations of AG, STK, and SHP1 or SHP2 form ternary complexes and promote ovule identity, most likely via a protein complex involving SHP1 and/or SHP2, STK, and SEP3 [37, 14]

Here, we report additional functions for *ABS* and *SHP1/SHP2* in ovule development, integument formation, endosperm development, and seed coat differentiation, and we further elucidate the genetic interactions between gynoecium and seed expressed MADS-box genes. Throughout the manuscript, we refer to the gene name *ABS* [3] whereas the name *tt16* refers to the knock-out mutant of the *ABS* gene [27].

## Results

### *ABS*, *SHP1* and *SHP2* are required for seed coat differentiation and seedling development

Previously, it was shown that the *ABS* function in endothelium development is masked by genetic redundancy with *STK* [18]. We produced the triple mutant *tt16-1 shp1-1 shp2-1* (called *tt16 shp1 shp2* below) to assess if *ABS* also genetically interacts with other MADS-box genes, such as *SHP1* and *SHP2* which share their domain of expression at least in part with *ABS* [38, 39].

**Fig 1:**
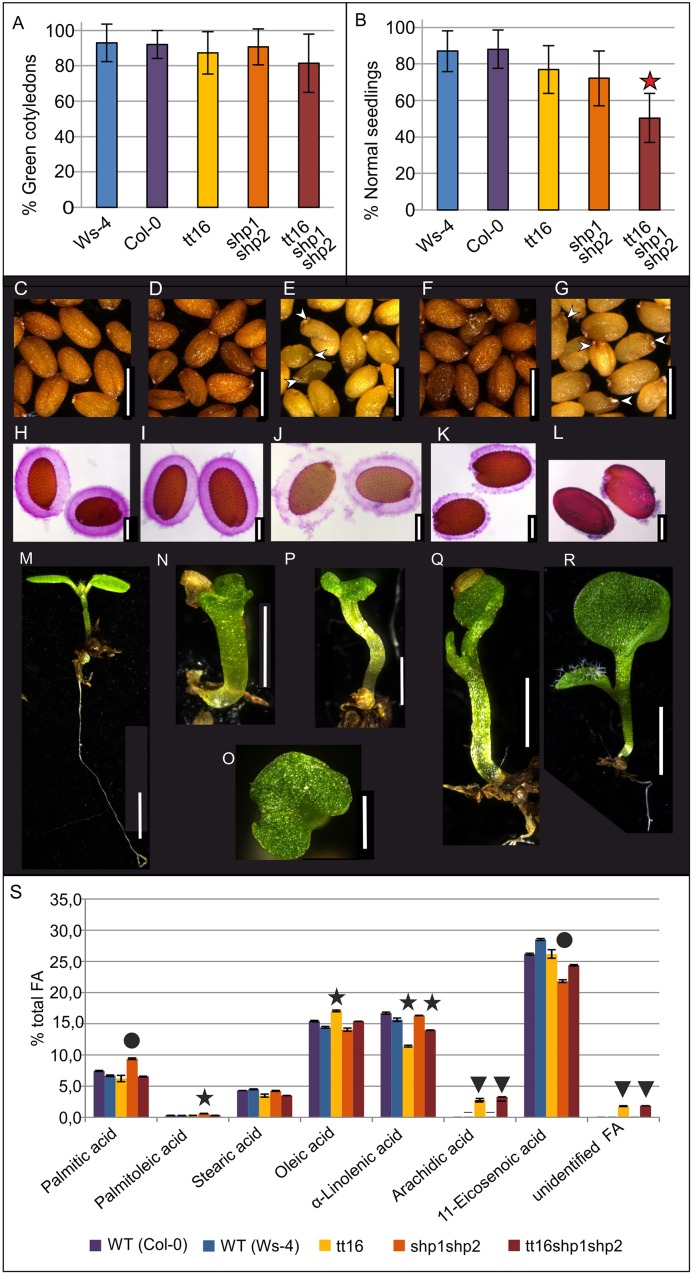
Defects in seedling development, mucilage release, and seed oil content analysis in Ws-4 and Col-0 wild type plants and in *tt16*, *shp1 shp2*, and *tt16 shp1 shp2* mutants. (A), cotyledon greening assay of seedlings grown on soil with appearance of green cotyledons scored as criterion for the viability. (B), proportion of seedlings germinated on soil without observed developmental defects, star indicates significant difference to Ws-4 (student’s t-test, p ≤ 0.05). Seed morphology is shown of Ws-4 (C), Col-0 (D), *tt16* (E), *shp1 shp2* (F), and *tt16 shp1 shp2* (G) seeds. Arrowheads in (E) and (G) indicate malformed seeds. Ruthenium Red assay was carried out to observe the quantity and distribution of mucilage on hydrated seeds of Ws-4 (H), Col-0 (I), *tt16* (J), *shp1 shp2* (K), and *tt16 shp1 shp2* (L) seeds. (M)-(R), one week old seedlings of the Col-0 (M), and *tt16 shp1 shp2* mutants germinated on soil (N)-(R) illustrating the different types of abnormal seedling development observed, such as short roots, fused cotyledons, malformed hypocotyls, and a reduction in size of the cotyledons. (S), comparison of mature seed fatty acid composition between wild type (WT) *A*. *thaliana* accessions (Col-0, Ws-4) and the mutant genotypes *tt16*, *shp1 shp2* and *tt16 shp1 shp2*, respectively. Significances were tested with a Post-Hoc-test. Circles: p<0.001 when compared to all other plant lines; stars: p<0.0001 when compared to all other plant lines; arrowheads: p<0.0001 when compared to the wild types or to the *shp1 shp2* mutant. Scale bars in C, D, E, F, G, N: 500μm; H, I, J, K, L: 200μm; M: 2mm; O, P, Q, R: 1mm.

First we observed the viability of the seedlings for the wild type Ws-4 (as *tt16-1* is an INRA-Versaille line mutant and Ws-4 is the parental line for this mutant population) and Col-0 (as the *shp1 shp2* mutant is in Col-0) and mutant seeds (*tt16*, *shp1 shp2*, and *tt16 shp1 shp2* obtained by crossing) which detached from the fruit since the seeds were sieved after harvesting. While the cotyledon greening assay on soil was similar for the genotypes tested (Fig 1A), serious developmental defects were observed in the *tt16 shp1 shp2* triple mutant seedlings, when compared to all other genotypes (Fig 1B).

We then observed the seed morphology of the mutants and two wild type accessions (Fig 1C–1G, details are shown enlarged in S1A–S1E Fig). The wild type genotypes and the *shp1 shp2* seeds were brown and not fully expanded seeds were only very rarely observed (Fig 1C, 1D and 1F). The seeds of the *tt16* mutant plants were straw-colored (Fig 1E) with a darker chalaza (S1C Fig), as reported previously by Nesi et al. 2002 [27]. Seeds from the *tt16 shp1 shp2* mutant showed a similar coloration (Fig 1G and S1E Fig). However, both, *tt16* and the triple mutants produced many seeds that were not fully expanded and deformed suggesting that endosperm filling or embryo expansion may be impaired when *ABS* gene function is compromised. Interestingly, this aspect of the *tt16* phenotype cannot be compensated by the simultaneous loss of *SHP1* and *SHP2*.

The first step during the germination process is rehydration, which occurs when seeds are exposed to water. Upon imbibition the outer layer of the seed coat expands and the mucilage it produces is also hydrated. Several intertwined developmental and biochemical processes are required for mucilage production. Firstly, the integuments need to differentiate in the ovule, providing the cells that later constitute the testa. The epidermal cells then produce thick secondary cell walls, synthesize and secrete pectins and provide transcription factors and enzymes for maturation and linking of the pectins to the epidermal cells by cellulose (for review see North et al., 2014 [40]). We used Ruthenium Red staining to characterize the mucilage production and adherence (Fig 1H–1L, see also S1F–S1J Fig for a close-up of the seed coat of imbibed seeds). The two wild type ecotypes Ws-4 and Col-0 showed a thick uniform layer of mucilage that adhered firmly to the seed coat (Fig 1H and 1I). Seeds of *tt16* produced mucilage that expanded to the same thickness as in the wild types, but the mucilage detached easily from the seed coat and was less intensely stained. This suggests that the polysaccharide matrix is less dense and less well connected to the epidermis (Fig 1J). This result contrasts to a previous report by Dean et al. (2011) [41], who did not observe a difference in mucilage quantity between Ws and *tt16* seeds. However, the growth conditions were different; the plants were grown in continuous light in the experiments by Dean et al. (2011) [41] while we grew them in long day conditions. The seeds of *shp1 shp2* developed a thinner mucilage layer with an uneven distribution of pectins (Fig 1K), suggesting that mucilage is produced but does not expand properly or is not fully released from the epidermal cells. However, mucilage adherence to the epidermis did not seem to be affected (Fig 1K). The triple mutants showed only small patches of mucilage remaining on the seed, indicating additive effects of the *tt16* and *shp1 shp2* mutations and consequently both a reduction in production and adherence of mucilage (Fig 1L). These observations indicate that not only the endothelium layer is affected by the *tt16* mutation, but also the epidermal cell layer of the seed coat. Furthermore they suggest that epidermal seed coat development may be also affected in the *shp1 shp2* mutant.

A morphological analysis of the seedlings was subsequently performed to describe the abnormalities that were observed in around 50% of the triple mutant seedlings (Fig 1B). Around 90% of wild type seedlings and more than 70% of *tt16*, and *shp1 shp2* seedlings showed a root of around 8 to 9 mm in length, two fully expanded cotyledons and the first set of true leaves emerging from the shoot apical meristem (SAM) five days after stratification (Fig 1M). 50% of *tt16 shp1 shp2* seedlings showed defects in development, with a lack of properly developed roots, defective cotyledon expansion and a frequent inability to develop true leaves (Fig 1N and 1P). In most cases the small roundish cotyledons are partially fused (Fig 1N–1P), and a large portion of the abnormal seedlings (~ 70%) failed to develop roots or true leaves and died within 12 days after stratification. Even if the seedlings survived and were able to produce leaves (Fig 1Q and 1R), they were smaller in size and also lagged behind the development of the wild types, *tt16*, and *shp1 shp2* plants throughout their life.

In many cases, fused organs (either failed to separate or fused together later in development) can be observed in the malformed seedlings (Fig 1O), and this may point towards a differential regulation of proteins involved in fatty acid biosynthesis in wild type and the mutants [42], more specifically in the fatty acid composition that specifies the properties of the embryo’s cuticula. However, Focks and Benning (1998) [43] reported that a severe reduction in seed oil content and a change in fatty acid composition caused by the *wrinkled1* mutant do not alter plantlet growth and morphology.

S1 Fig compares the fatty acid composition in the seed storage oil of the wild type and mutant genotypes. The *tt16* mutant showed elevated oleic acid (C18:1) content with simultaneously decreased alpha-linolenic acid (C18:3). This supports previous results from Deng et al. (2012) [18], who described a reduction in fatty acid desaturase (FAD) gene expression in *Brassica napus tt16* RNAi lines. Alterations in fatty acid desaturation may also explain the slight increase in arachidic acid (C20:0) and another unidentified fatty acid in the *tt16* and *tt16 shp1 shp2* mutants. Interestingly, the observed changes in oleic acid/alpha linolenic acid composition were more pronounced in the *tt16* single mutant compared to *tt16 shp1 shp2*, consistent with the compensatory effect of the triple mutant that was observed for other seed development traits (see below).

As the seedlings did not show a uniform phenotype, *ABS*, *SHP1*, and *SHP2* may not be involved in establishing embryo architecture. Instead the three genes may be involved in nutrient supply to the embryo, resulting in malnourished seedlings when their function is lost. Alternatively, the malformation could be caused by mechanical stress of the embryos not being able to increase in size due to a seed cavity too small in size.

### *ABS*, *SHP1* and *SHP2* act in synchronization of ovule/seed maturation and in endosperm development

We and others (Fig 1; [27, 28, 18]) have shown that *ABS* is required for proper seed development. However, previously published work describing the function of *ABS* was limited to the formative divisions of the inner integument, the development of the endothelium cell layer and regulation of anthocyanin biosynthesis. The data presented in Fig 1 already indicate that this may not draw the complete picture of *ABS* function, as we observe defects in mucilage production, seed expansion and seedling development in the *tt16 shp1 shp2* mutants. These data suggest a function for *ABS*, *SHP1*, and *SHP2* in these tissues. To learn which developmental defects lead to the consequences presented in Fig 1, we thoroughly analyzed seed coat, endosperm, and embryo development in *tt16*, *shp1 shp2* and in *tt16 shp1 shp2* mutant lines by observing cleared seeds of different developmental stages.

**Fig 2:**
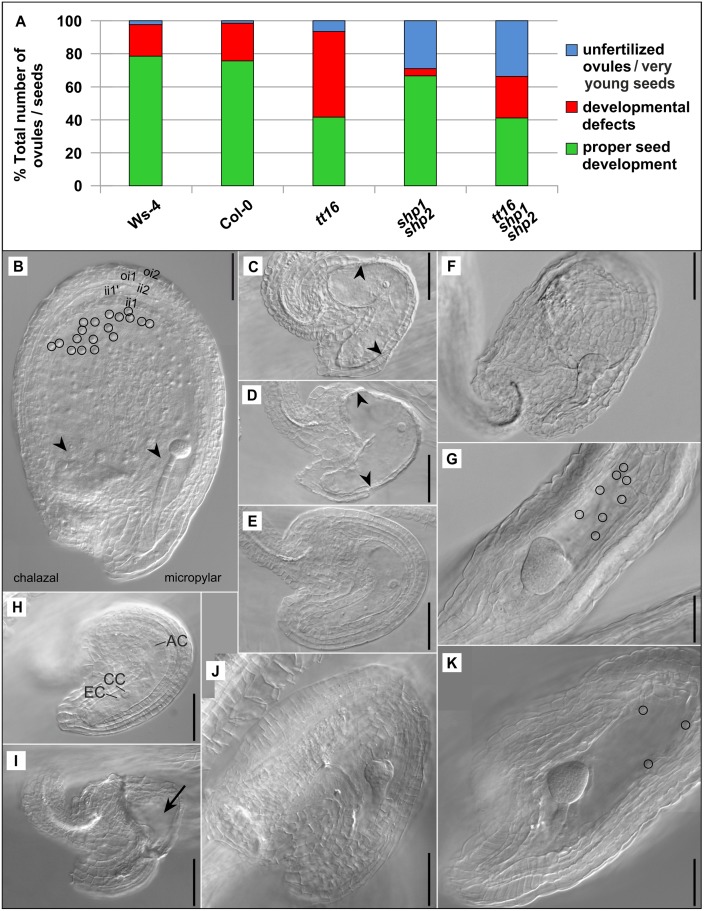
Seed developmental defects of Ws-4 and Col-0 wild type plants and of *tt16*, *shp1 shp2*, and *tt16 shp1 shp2* mutants. (A), amount of unfertilized ovules/very young seeds (classified as stage 3-VI of megagametogenesis or post-fertilization stage 4-I according to Schneitz et al., 1995 [44]), ovules/seeds with developmental defects, and properly developed seeds given in percentages of the total number of ovules/seeds found in stage 16 and stage 17A siliques (stages defined according to Ferrándiz et al., 1999 [45]) containing octant, globular or early heart embryos. For Ws-4, n = 257; Col-0, n = 193; *tt16*, n = 463; *shp1 shp2*, n = 234; *tt16 shp1 shp2*, n = 202. (B), properly developed seed of a Ws-4 plant with a globular embryo. The micropylar and chalazal endosperm appears intact (arrowheads) and the syncytial peripheral endosperm contains many nuclei (e.g. circles). The seed coat develops from the two layers of the outer integument (oi2 and oi1) and the three layers of the inner integument (ii2, ii1´, and ii1). The innermost endothelium layer (ii1) is composed of thick cells with an almost isodiametric shape. Properly developed seeds of Col-0 plants appear similar. (C) to (K), unfertilized ovules/very young seeds and malformed seeds found in stage 16 and stage 17A siliques harboring octant, globular or early heart embryos. Developmental defects in Ws-4 (C) or Col-0 (D) where the lack of a properly developed embryo and shrinkage of the integument or seed coat layers (between arrowheads) indicates that these ovules/seeds are most likely destined to abort. (E), Col-0, rare case of an unfertilized ovule/very young seed with a normal appearance. (F) and (G), small-sized and slender *tt16* mutant seeds harboring embryos at globular (F) and early heart stage (G), but containing only little (G, circles) or no peripheral endosperm (F). The embryo in (F) appears mechanically squeezed, possibly due to its short suspensor and the small seed size. (H) shows an unfertilized ovule of normal appearance which were regularly found next to well-developed seeds in the siliques of *shp1 shp2* mutant plants. AC: antipodal cell; CC: central cell nucleus; EC: egg cell nucleus. (I), a presumably unfertilized and aborted ovule of a *tt16 shp1 shp2* triple mutant shows a malformed embryo sac (black arrow) and shrunken integument layers. (J), small-sized *tt16 shp1 shp2* seed containing an early globular embryo with a slight defect in the protoderm, but no endosperm. (K), slender seed of a *tt16 shp1 shp2* plant with well-developed micropylar endosperm surrounding the early heart stage embryo, but only few peripheral endosperm nuclei / cells directly attached to the seed coat (circles). This seed resembles the seed of the *tt16* mutant in (G). Scale bars in (B)-(K) are 50 μm.

Seed development is a highly complex process which starts with ovule development and fertilization, followed by coordinated embryo, endosperm, and testa formation. This long-term growth process requires a constant flow of nutrients from the carpel marginal tissue. A failure in any of these subordinate processes can result in developmental defects of the seed and embryo. To characterize details of the developmental defects in which the *tt16* and *tt16 shp1 shp2* mutants deviate from that of wild type seeds, we closely observed ovules/seeds in stage 16 to 17A siliques in which properly developing seeds with embryos at octant, globular or heart stages are found (Fig 2A). Ws-4 and Col-0 wild type seeds were rather similar, with less than 3% of unfertilized ovules/very young seeds observed (classified as stage 3-VI of megagametogenesis or post-fertilization stage 4-I according to the morphological criteria defined by Schneitz et al., 1995 [44]; see below) and around 77% of properly developed seeds, leaving approximately 21% of seeds that showed defects in embryo or endosperm development. In the *tt16* mutant, the number of unfertilized ovules/very young seeds was increased to around 6.5%, but the number of seeds showing developmental defects in embryo or endosperm development was increased to over 50%. Conversely, 29% of the ovules in *shp1 shp2* mutants remained unfertilized or arrested in a very young post-fertilization stage, but in most fertilized ovules, the development of the embryo and endosperm commenced normally and almost 67% of the seeds developed normally. In the triple mutant, the number of unfertilized ovules/very young seeds was only slightly higher than in the *shp1 shp2* double mutant, ranging around 34%. Another 25% of seeds showed abnormal embryo or endosperm development, leaving only around 41% normally developed seeds.

A thorough analysis of Ws-4 and Col-0 ovule and seed development is documented in S2 Fig. The typical defects observed in the two wild type ecotypes (Ws-4 and Col-0) and in the mutant lines are shown in Fig 2C–2K in comparison to a properly developing Ws-4 seed Fig 2B. The correctly developing wild type seeds exhibited intact embryos at globular stage surrounded by endosperm nuclei and the five-layered testa composed of cells in the typical pattern: the two inner cell layers included large roundish cells while the outer three layers were made of thinner, more rectangular cells (Fig 2B for Ws-4 and S2E and S3A Figs for Col-0, later stages of both wild types are shown in S2F–S2H Fig). Abnormal development of wild type seeds was mostly accompanied by the lack of an embryo, indicating early abortion of unfertilized ovules or very young seeds (Fig 2C and 2D) while unfertilized ovules/very young seeds with a normal appearance were only rarely found (Fig 2E).

Developmental defects were observed in the *tt16* mutant seeds. In many cases the endosperm failed to develop completely (Fig 2F) or only the peripheral endosperm was significantly reduced or missing, whereas the development of the embryo (S5 Fig) and the chalazal and micropylar endosperm seemed largely unaffected, at least until the globular stage. As seen in Fig 2F, the regularly layered cells of the seed coat often are missing, with the seed instead showing an unstructured testa with irregularly shaped cells. The small seed size may indicate premature abortion (compared with Fig 2B for a WT seed at respective developmental stage). In other *tt16* seeds, the embryo continued to develop normally and some endosperm was formed (Fig 2G), most likely resulting in shriveled seeds at maturity. Unfertilized ovules/very young seeds with a normal appearance were rarely observed in the *tt16* mutant siliques (S3B Fig). As observed for 0.4 to 2% of all investigated plant lines’ seeds including the wild types, also the *tt16* mutant embryos showed irregularities in protoderm development (S5B–S5D Fig; see also Fig 2J and S5G and S5H Fig for triple mutant embryos). Sporadically (between 0.3 and 1.5% of the seeds), conspicuously short suspensors were observed, which may cause spatial and mechanical problems during further embryo growth (Fig 2F and S5E and S5F Fig; see also S5H and S5I Fig for triple mutant embryos). Taken together, however, these results demonstrate that *ABS* has no prominent role in embryo development, but is involved in endosperm formation. Reduction in the number of peripheral endosperm nuclei suggests arrested endosperm nuclear divisions, but defects in the migration of endosperm nuclei can also not be ruled out.

Stage 16 and 17A siliques of the *shp1 shp2* mutants showed a large fraction of presumably unfertilized ovules in which the embryo sacs were not markedly curved and possessed antipodal cells besides only one (fused) central cell nucleus, indicating stage 3-VI of megagametogenesis (Fig 2H; [44]). Whether the central cell nucleus had already fused with a sperm nucleus to form the endosperm nucleus in the post-fertilization stage 4-I could not always be ruled out, but nuclear endosperm development had certainly not been initiated and stage 4-II was not reached.

The *tt16 shp1 shp2* triple mutant showed additive effects of the mutant phenotypes and had many unfertilized ovules/very young seeds as well as many seeds with severe developmental deficiencies (Fig 2A and 2I–2K). In contrast to the wild types, lack of the embryo was only seldom observed (Fig 2I), but the fertilized ovules regularly resulted in developing seeds with a complete lack of endosperm. Fig 2J shows such a structure, with an embryo at early globular stage that developed without surrounding endosperm in a seed that failed to expand (cf. S3A Fig for the respective wild type seed). In several cases the chalazal and micropylar endosperm was formed, but the peripheral endosperm developed to a much lower extent than in the wild types (Fig 2K, see S2F Fig for the respective wild type seed). In the milder form of the triple mutant phenotype, the seed cavity has a normal size, although the quantity of endosperm still fails to reach that of the wild type. Detailed images of endosperm content in the triple mutant seeds with embryos at early globular stage and heart stage of weaker phenotypes are shown S3C–S3E Fig showing well developed micropylar and chalazal endosperm but missing peripheral endosperm.

In summary, the presented data indicate that *ABS* is important for endosperm development and that *SHP1* and *SHP2* are required for timely ovule/seed development and/or fertilization. However, in the triple mutant we observed that the number of unfertilized ovules/very young seeds remained similar to the *shp1 shp2* double mutant and endosperm developmental defects were those seen in the *tt16* mutant suggesting no genetic interactions of *ABS* and *SHP1/SHP2* in these aspects of ovule and seed development.

### *ABS* regulates formative divisions and cell shape of inner integument cell layers and *SHP1* and *SHP2* act antagonistically to *ABS* in these developmental processes

The *A*. *thaliana* inner seed coat layer ii1 is the innermost maternal layer of the seed. It differentiates into the endothelium which mediates nutrient delivery to the endosperm and embryo. In the following, the term “endothelium” is reserved for the innermost cell layer (ii1) of the wild type seed coats. Because we cannot be sure that the innermost seed coat layer of the mutants develops into an endothelium, we refrain from using the term endothelium, but use the term ii1 for the innermost seed coat layer and ii1´ for the second innermost layer [46] in all the mutants used.

**Fig 3:**
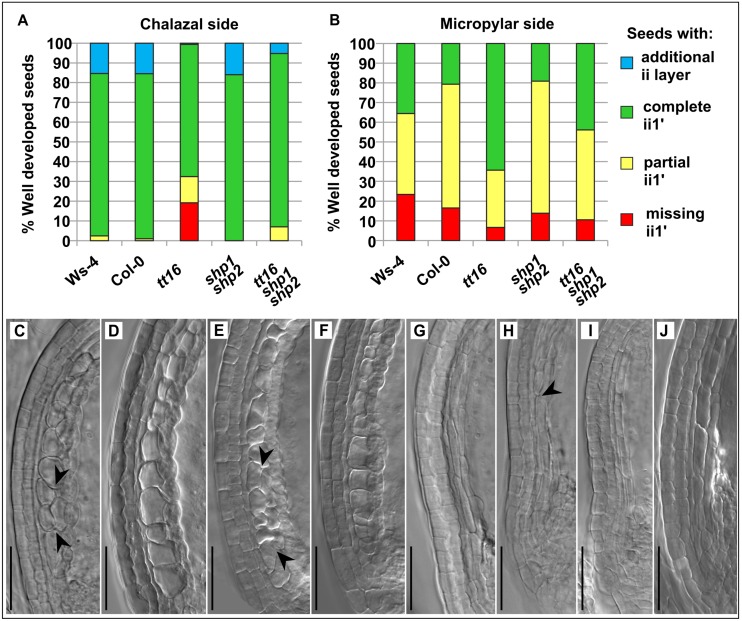
Quantitative analysis of the presence of the ii1´ seed coat layer in well-developed seeds of stage 16 and 17A siliques harboring globular to heart staged embryos. (A) and (B), percentages of seeds with, without, and with only partially developed ii1´ layer, or with a locally occurring additional fourth layer on the chalazal side (A) and on the micropylar side (B). For Ws-4, n = 124; Col-0, n = 97; *tt16*, n = 151; *shp1 shp2*, n = 94; *tt16 shp1 shp2*, n = 57. (C)-(J), documentation of the seed coat layer anatomy at the chalazal side of well-developed seeds in the wild type and mutant lines. In Ws-4 (C) and Col-0 (D) wild type seeds, the ii1´ cell layer usually is complete and reaches up to the chalazal end (additional incomplete fourth cell layer is formed in some cells, between arrowheads in C). ii1 and ii1´ cells have an almost isodiametric shape (C, D), with ii1´ cells being highly vacuolated whereas the ii1 cells (endothelium) appear densely packed. The *shp1 shp2* seed coat may show an incomplete, extra layer of thick, vacuolated cells adjacent to the ii1´ layer (between arrowheads in E) or the normal five layers (F). (G), *tt16* seed coat with five complete cell layers showing thin elongated cells in the inner two layers (cf. also S3F Fig). (H), *tt16* seed coat, partially missing the ii1´ layer which does not reach the chalazal end (below arrowhead) and (I), complete lack of the ii1´ layer at the chalazal side of a *tt16* seed coat. (J), *tt16 shp1 shp2* mutant seed coat with five complete cell layers but the two inner seed coat layers are composed of thin elongated cells lacking dense cytoplasm. Scale bars in (C)-(J) are 50 μm.

Because the ii1 cells in *tt16-1* plants show cell division defects, were longer, flatter and more vacuolized than in wild type, and the endothelium was missing completely in *tt16-6 stk* mutants [27, 28, 18], we thoroughly analyzed the growth pattern of the endothelium/ii1 layer whose periclinal divisions give rise to the neighboring ii1’. We investigated well developed seeds with globular to heart staged embryos in wild type plants of two ecotypes, Ws-4 and Col-0, and in the three mutant lines to better understand the defects observed in the *tt16* mutants and to learn if *SHP1* and *SHP2* also have a role in inner seed coat development. Fig 3A and 3B (and S4A–S4J Fig) give a quantitative overview of the inner seed coat layer anatomy in the different plant lines, while representative examples are shown in Fig 3C–3J and S4K–S4V Fig.

For a clearly laid-out documentation of the observations we divided the seed coat in the chalazal side (starting from the chalazal towards the distal end) and the micropylar side (starting from the micropylar to the distal end) (S2E Fig), although both sides originate from the curving zone at the abaxial side of the ovule (S2C and S2D Fig). The two wild type ecotypes Ws-4 and Col-0 showed almost identical layer patterns at the chalazal side of the inner seed coat. More than 82% of the seeds had a complete ii1´ layer reaching from the distal end of the seed to the chalazal end (Fig 3A). Around 15% of the seeds showed an incomplete additional cell layer between the presumed endothelium (ii1) and the ii1’ layer. In the *tt16* mutant, the ii1 layer had often not divided properly, so that 19% of the seeds lacked the ii1´ layer at the chalazal side, over 13% showed only a partial ii1´ layer which failed to reach down to the chalazal end, and an additional cell layer was observed only once (0.7%). The ii1´ layer of the *shp1 shp2* mutant appeared as in the wild type ecotypes. Interestingly, in the *tt16 shp1 shp2* triple mutant, all seeds had at least a partial ii1´ layer at this developmental stage (missing ii1’ occurred seldom in younger seeds with octant stage embryos as shown in S4E Fig). 88% of the seeds showed a complete ii1´ layer at the chalazal side, and 5% had an additional cell layer suggesting that the absence of *SHP1* and *SHP2* gene function partially reliefs the defects caused by the mutation in *ABS*. An effect of the distinct genetic backgrounds of the *tt16* single (i.e. WS-4) and triple mutant line (i.e. mix of WS-4 and Col-0) can be excluded based on our data, as cell layer development was identical in both ecotypes. Details on the time course of the formative divisions in the inner seed coat can be deduced from S4A–S4E Fig which shows that the ii1´ layer proliferation does not proceed significantly during early postfertilization development at the chalazal side and that the *tt16* mutant phenotype can already been detected at very early stages of seed development.

The pattern of formative divisions in the inner seed coat was different at the micropylar side where development of the ii1´ layer continued for some time in all plant lines during early seed development (S4F–S4J Fig), and minor variations were found even between the wild type accessions (Fig 3B). The endothelium had divided properly and the ii1´ layer reached completely down to the micropylar end in only 35% and 21% of the seeds in Ws-4 and Col-0, respectively, but the ii1´ layer was absent in 23% and 16%, respectively. Interestingly, only 7% of the *tt16* mutant seeds completely lacked the ii1´ layer at the micropylar side, but around 64% had a complete ii1´ layer, which develops at earlier developmental stages than in the other plant lines, including the Ws-4 wild type and the triple mutant to a minor extent (S4H and S4J Fig). While *shp1 shp2* mutant seeds showed almost the same ratios of ii1´ layer present/absent than the Col-0 wild type (Fig 3B), the layer structure at the micropylar side of the inner *tt16 shp1 shp2* mutant seed coat represented an intermediate of the *tt16* mutant seeds on the one hand and the wild type and *shp1 shp2* double mutant seeds on the other hand. The triple mutant showed 20% fewer seeds with a complete ii1´ layer (44%) than the *tt16* single mutant but more than all other plant lines. Also, the percentage of triple mutant seeds lacking the ii1´ layer at the micropylar side (11%) is between the *tt16* mutant and the other plant lines. This suggests again that *SHP1 SHP2* and *ABS* may act antagonistically, although in this case effects caused by the distinct genetic background of the mutants must also be taken into account. Taken together these results suggest that *ABS* promotes the formative cell divisions of the endothelium (or ii1) at the chalazal side, but inhibits them at the micropylar side. And while the *shp1 shp2* mutant seeds appear like (Col-0) wild types, the absence of *SHP1* and *SHP2* is able to partially rescue the effects of *ABS* loss of function.

**Fig 4:**
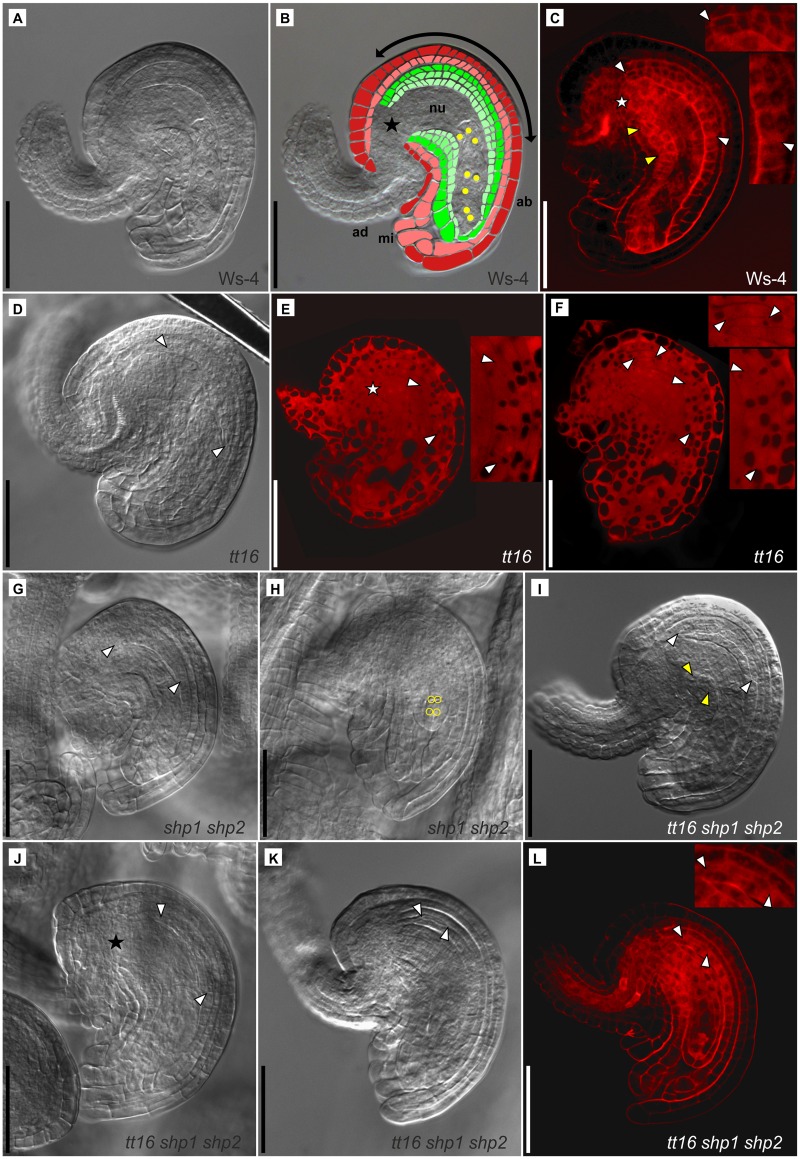
Ovule anatomy of Ws-4 wild type plants and developmental defects of *tt16*, *shp1 shp2*, and *tt16 shp1 shp2* mutant ovules of floral stage 12 (stages defined after Smyth et al., 1990) which were classified as almost mature and fully mature ovules at late stage 3-V or stage 3-VI (stages defined after Schneitz et al., 1995 [44]). (A)-(C), Ws-4 ovules, DIC micrograph (A) and colorized version of the same image (B) showing ovule at stage 3-V whose eight-nuclear embryo sac (yellow dots) has undergone cellularization. Labels are as follows: outer integument 2 and 1 (oi2, dark red; oi1, light red), inner integument 2, 1´, and 1 (ii2, dark green; ii1´, green; ii1, light green); ad, adaxial side; ab, abaxial side; double headed arrow, curving zone; mi, micropyle; nu, nucellus; star, chalazal end). Ovule anatomy is similar in Ws-4 and Col-0. (C), CLSM micrograph of a Ws-4 ovule stained with propidium iodide also illustrates the normal occurrence of the ii1´ layer at the adaxial (between yellow arrowheads) and abaxial side (between white arrowheads). For the abaxial side, the start of the ii1´ layer directly at the chalazal end (star) and the end of the ii1´ layer are shown enlarged in the respective insert (white arrowheads). (D), normal development of the abaxial ii1´ layer of a *tt16* mutant ovule (between arrowheads). (E), *tt16* ovule, where several cells at the chalazal end (star) of the abaxial ii1 layer fail to undergo periclinal divisions, but formation of the ii1´ layer (between arrowheads and see insert) starts further down towards the micropylar end. (F), patchy development of the ii1´ layer occurring at the abaxial side of *tt16* mutant ovules. Periclinal divisions start at two independent positions (between white arrowheads, enlarged in the inserts). (G), normal development of the ii1´ layer (between arrowheads) at the abaxial side of a *shp1 shp2* ovule which contains an almost mature embryo sac. (H), young ovule at an earlier stage of megagametogenesis (3-IV, [44]) found besides almost mature and fully mature ovules in the same *shp1 shp2* pistil. The nuclei of the four-nuclear embryo sac are marked with yellow circles. (I), normal development of the abaxial ii1´ layer of a *tt16 shp1 shp2* ovule (between white arrowheads). At the adaxial side, the first two ii1´ cells formed by periclinal divisions of ii1 at the chalazal end (between yellow arrowheads). (J), the formation of the ii1´ layer at the abaxial side of this *tt16 shp1 shp2* ovule fails to start at the chalazal end (star), but starts a few cells further down towards the micropylar end (between arrowheads). (K) and (L), development of some *tt16 shp1 shp2* ovules also lags behind in comparison to other ovules of the same pistil. Formation of the abaxial ii1´ layer has only recently begun and only one or two ii1 cells have already divided at the chalazal end (between arrowheads in Figs (K) and (L), and shown enlarged in the insert of (L)). The *tt16 shp1 shp2* ovule shown in (L) is at an earlier stage of megagametogenesis than the almost mature ovules found in the same pistil. Scale bars in (A)-(L) are 50 μm. Clearing of the propidium-iodide stained CLSM samples was performed with chloral hydrate in (C) and (L), and with benzyl benzoate/benzyl alcohol in (E) and (F). Ovules of at least five pistils collected from different plants were observed for every plant line.

Further, we quantitatively analyzed also the morphology of the ii1’ layer and the endothelium (or ii1) cells, as in many cases defects in the endothelium/ii1 layer coincided with defects in the neighboring ii1’ layer (S3F and S3G Fig, also Fig 3C–3J and S4K–S4V Fig and Fig 4K–4V). At the chalazal side, wild type seeds of the Ws-4 and Col-0 ecotypes generally produced endothelium and ii1’ layers made up of round cells as shown in Fig 3C and 3D, only rarely showing an incomplete, additional layer (Fig 3C). Only in 14% of the Ws-4 seeds and 3% of the Col-0 seeds which had an at least partially developed ii1´ layer, the endothelium and/or ii1’ layers were composed of thin cells at the chalazal side (S3F Fig). 24% of the *shp1 shp2* mutant seeds showed thin and elongated cells in the ii1 and/or ii1’ layers, but the cell morphology was roundish in the majority of seeds (76%; S3F Fig, Fig 3E and 3F), with an additional layer observed in rare cases (Fig 3E). In more than 95% of the *tt16* mutant seeds, however, the ii1 and/or ii1’ layer were composed of thin elongated cells at the chalazal side (S3F Fig, Fig 3G, 3H and 3I), which corroborates the findings by Nesi et al. (2002) [27] and Debeaujon et al. (2003) [28]. Remarkably, 28% of the seeds developed normal morphology of the ii1 and ii1’ cells when *tt16* is combined with *shp1 shp2* (S3F Fig). The inner seed coat layers of the triple mutant often had an intermediate cell shape which was elongated, but not as thin as in the *tt16* single mutant (Fig 3J).

On the micropylar side, the difference of the cell morphology in the inner seed coats was more pronounced between the two wild type accessions, with only 43% of the Ws-4 seeds but 71% of the Col-0 seeds showing round endothelium and ii1´ cells (S3G and S4K–S4P Figs). While the cell shape of the inner *shp1 shp2* mutant seed coat was similar to the Col-0 wild type (S3G and S4Q Figs), only 20% of the *tt16* seeds had round cells in the ii1 and/or ii1´ layer at the micropylar side (S3G, S4R and S4S Figs). And again, when the two mutants are combined in the *tt16 shp1 shp2* plants, their seeds ranged between the two individual mutant lines and closely resembled the Ws-4 wild type with around 45% of the seeds showing round ii1 and ii1’ cells the micropylar side (S3G and S4T–S4V Figs).

As far as can be deduced from the observation of cleared seeds, endothelium/ii1 cells of both wild types and the *shp1 shp2* double mutant showed a .showed remnants of a dense cytoplasmic content (Fig 3C–3F, S4K, S4L and S4N–S4Q Fig; with rare exceptions, S4M Fig) which is probably due to the relatively low degree of vacuolization and/or to pigment accumulation in this cell layer [4].

However, this feature was lacking in most ii1 cells of the single *tt16* mutant, and also in the triple mutant (Fig 3G–3J, S4R–S4V Fig) which corroborates the previous observation of a higher degree of vacuolization and compromised PA biosynthesis in the ii1 layer of the *tt16* mutant [27, 28].

In summary, our analysis shows that *ABS* directs formative cell divisions of the innermost seed coat layer on the chalazal as well as on the micropylar side. Moreover, *ABS* is also required for the final cell shape of the endothelium (or ii1) and its neighboring ii1’ layer. In contrast, *SHP1* and *SHP2* loss of function has hardly any influence on cell division pattern or shape of the inner seed coat layers. But in all cases observed, the periclinal cell division defects and cell shape alterations observed in the *tt16* mutant are at least partially restored by the additional loss of *SHP1* and *SHP2* gene function, except for the changes in cytoplasm density.

### Defects in ovule formation trigger inner seed coat layer malformation and delayed fertilization/seed development

The data presented in Fig 2A suggest a role for *SHP1* and *SHP2* in the synchronization of ovule/seed maturation and/or fertilization, while Fig 3 and S4 Fig show that *ABS* influences development of the inner seed coat layers. Because these layers are already formed as inner integument during ovule development, ovule morphology before fertilization of wild type and mutant lines was observed carefully. Ovules of Ws-4 and Col-0 plants develop in a very similar fashion (Fig 4A–4C, S2A–S2D Fig). In very young ovules, the two outer integument cell layers of the ovule encase the nucellus and the inner integument, and the two inner integument cell layers continue to grow towards the micropylar end (S2A Fig). Later, the outer and inner integuments surround the nucellus fully except for a small gap at the micropyle (S2B Fig). Both integuments are composed of two cell layers each (oi1, oi2, ii1, and ii2, terminology after Beeckman et al., 2000 [46]), but during gametogenesis, the innermost ii1 layer will divide further by successive periclinal divisions starting at the chalazal end and proceeding towards the micropylar end (S2B, S2C and S2D Fig). The newly formed layer, termed ii1’ is sandwiched between the ii1 and ii2 layers on the adaxial and abaxial side of the ovule. Fig 4A and 4B illustrate ovule morphology before fertilization by depicting the five integument cell layers and the eight-nucleate embryo sac. In Fig 4C, the position of the ii1´ layer is indicated to allow comparison with the mutant lines. We focused on the abaxial ovule side which is most decisive for future seed coat development, because the integument cells in the curving zone will undergo a massive expansion after fertilization and cover the surface of the developing seed (Fig 4B, S2D and S2E Fig; [46]).

In the *tt16* plants, ovules may develop normally (Fig 4D) but in accordance with the findings of Debeaujon et al. (2003) [28], often several of the ii1 cells at the outermost chalazal end of the abaxial ovule side fail to induce periclinal divisions to generate the fifth layer (Fig 4E). However, the position of the formative cell divisions was only spatially shifted towards the micropylar end and, with a few exceptions (Fig 4E), the overall length of the newly formed ii1´ layer was not affected. This spatial shift of ii1 cell divisions may cause the relocation of the ii1´ layer observed in developing *tt16* seeds (see Fig 3A and 3B). Sometimes, the ii1 division pattern was irregular (Fig 4F) which was not anticipated from the observation of the *tt16* seed coat phenotype. The initial gaps in the ii1´ layer must be filled in later developmental stages, as a patchy ii1´ layer phenotype was no longer observed in developing *tt16* mutant seeds. In contrast to the findings of Debeaujon et al. (2003) [28], we did not observe additional periclinal divisions forming a locally restricted fourth inner integument layer at the abaxial side of the *tt16* ovules. Also, the percentage of developing seeds with an additional inner seed coat layer was lower with the *tt16* mutant in comparison to the other plant lines (see Fig 3A). Analysis of *shp1 shp2* ovules shows that, unlike in *tt16* ovules, their morphology resembles wild type, but asynchronously developed ovules of different developmental stages are regularly found in one pistil (Fig 4G and 4H, see Fig 2A). *tt16 shp1 shp2* gynoecium mutants showed additive effects of the two mutant phenotypes: several ovules resembling those of the wild type were found (Fig 4I). However, many ovules showed a lack of formative cell divisions of the innermost integument layer at the chalazal end (Fig 4J), patchy initiation of periclinal divisions, or the arrangement of ovules with different developmental stages within the same pistil (Fig 4K and 4L).

In summary, the mutant phenotypes observed during seed development are already largely based on defects in ovule formation: highly asynchronous ovule development is indicative for the *shp1 shp2* mutants and *ABS* loss of function causes severe defects in the formative division pattern of the inner integument.

### Regulation of seed-related MADS-box gene expression by *ABS*, *SHP1*, and *SHP2* is fertilization and stage dependent

The expression of *ABS* has been elucidated only partially [47, 18, 27, 48]. We thus performed GUS staining assay for which 3.5 kb upstream of the transcription start site, and *ABS* exons and introns were fused to the GUS coding region and the GUS staining pattern was observed.

**Fig 5:**
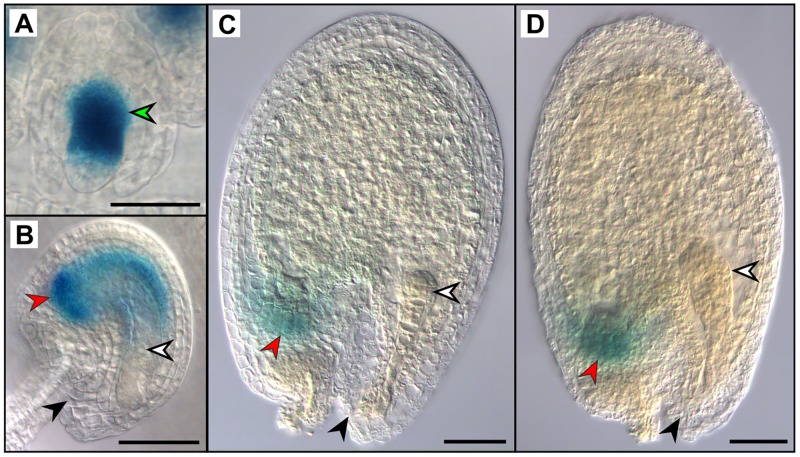
GUS staining assay of pABS::ABS:GUS. Young ovules in buds of stage 10 (A), in unfertilized ovules in stage 13 flowers (B), and in seeds from siliques at stage 16 to 17A harboring globular embryos (C) and heart stage embryos (D) were analyzed. Green arrowhead: staining in the nucellus and inner integument; black arrowheads: micropylar end; red arrowheads: chalazal endosperm; white arrowheads show the egg cell (B) and embryo (C, D); Scale bars: 50μm.

In stage 10 buds, when the embryo sac has not formed yet, GUS staining in the *pABS*::*ABS*:*GUS* line was observed in the nucellus and, albeit weaker, in the inner integument (Fig 5A). In mature ovules of stage 13 flowers just before fertilization, *pABS*::*ABS*:*GUS* staining was observed in the inner integuments and at the chalazal end of the ovule while the micropylar end remained unstained (Fig 5B), resembling the *in situ* hybridization pattern of *ABS* shown in Mizzotti et al. (2012) [18]. After fertilization, when the embryo is in the globular stage, expression decreased and GUS staining was visible only in the region of the chalazal endosperm of the *pABS*::*ABS*:*GUS* line (Fig 5C) where it remains restricted also when the embryo is at heart stage (Fig 5D). Even at later developmental stages (data not shown), GUS staining was never observed in the fruit outside the developing seeds. Our results deviated slightly, only after fertilization, from the ones published by Xu et al. (2016) [48] as a shorter promoter fragment of ABS (1,6 kb) was used to drive, together with the genomic locus, the GUS expression in their work.

We were then interested in elucidating the genetic interactions between *ABS* and *SHP1/SHP2* as previous work has shown that their expression patterns overlap [49, 50] and because our data suggest a genetic interaction between *ABS* and *SHP1/SHP2* (Fig 3 and S3F, S3G and S4 Figs).

**Fig 6:**
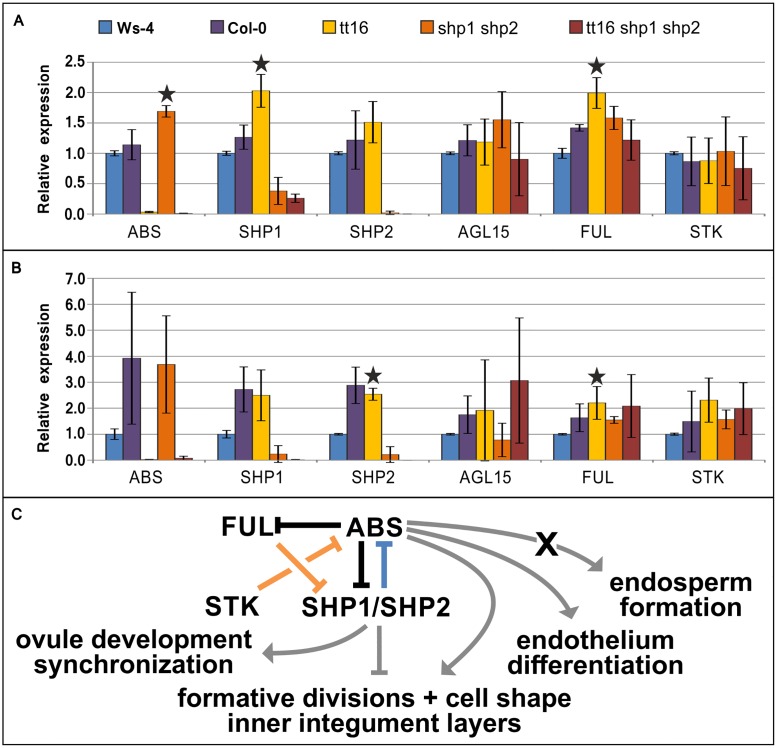
Relative expression of the MADS box genes *ABS*, *SHP1*, *SHP2*, *AGL15*, *FUL*, and *STK* in Ws-4, Col-0, *tt16*, *shp1 shp2*, and *tt16 shp1 shp2* analyzed by qRT-PCR. (A), gene expression in floral buds, (B), expression in siliques of stage 17A. Bars indicate standard deviations, stars indicate statistically significant difference (p < 0.05, tested by the one-tailed t-test) in expression, expression in *tt16* plants was compared to Ws-4, expression in *shp1 shp2* was compared to Col-0 and expression in *tt16 shp1 shp2* was compared to both wild types. (C), simplified model of genetic interactions between *ABS*, *SHP1*, *SHP2*, *FUL*, and *STK* (this work and [45, 52, 53]). A blue line indicates genetic interaction in floral buds, an orange line indicates regulation at stage 17A, a black line regulation at both stages of development, and a grey line indicates a gene´s impact on a morphological process. X symbolizes the temporal gap between expression and phenotypical effect.

For quantification of expression and the identification of additional putative genetic interactors of *ABS*, *SHP1*, and *SHP2*, we then selected two more MADS box genes for qRT-PCR analysis: *FRUITFUL* (*FUL*), a gene that is known to repress *SHP1* and *SHP2* [36] and *AGAMOUS-Like 15* (*AGL15)*, which is known to act in embryogenesis and seed development [51]. The expression levels of these six MADS box genes were observed by qRT-PCR in stage 1–12 floral buds (Fig 6A) and stage 17A siliques, when all floral organs have dehisced and the siliques are ~1 cm in length (Fig 6B).

Unexpectedly, the expression analysis shows different expression levels of these highly conserved MADS-box genes in Ws-4 and Col-0 wild types. For some of the genes tested this was observed in both tissues, but more pronounced in stage 17A siliques (Fig 6A and 6B). While this may suggest sequence dissimilarities in cis-regulatory regions, this limits observations, as the *tt16-1* mutant is an INRA-Versaille mutant line whose parental line is Ws-4, the *shp1 shp2* mutant is in Col-0 and the triple mutant is a mix of both ecotypes. We thus compare the expression in *tt16* lines to Ws-4, the expression of *shp1 shp2* to Col-0 and the *tt16 shp1 shp2* expression to both wild type accessions. Our analysis does not allow the distinction between direct and indirect regulation of target genes, in the following section we thus describe activation/repression with keeping in mind that the interaction can be direct or indirect.

In the floral buds (Fig 6A), the expression of *SHP1*, but not *SHP2* increased in the *tt16* mutant suggesting that *ABS* negatively regulates *SHP1* expression, and in the *shp1 shp2* double mutant, *ABS* expression is up-regulated. This suggests mutual negative regulation between *ABS* and *SHP1/SHP2*. The expression level of *AGL15* and *STK* was not affected significantly by the loss of neither *ABS*, nor *SHP1*/*SHP2*, but *FUL* expression increased significantly in *tt16* mutants.

Interestingly, the genetic interactions between the transcription factors changed between pre- and post-fertilization. When seed development is well under way in stage 17A (Fig 6B), *ABS* no longer shows significant increase in expression in *shp1 shp2* mutants and, at this developmental stage, *SHP2* but not *SHP1* is up regulated in the *tt16* mutant. This suggests individual regulatory differences between SHP1 and SHP2 which may or may not result in functional differences between these genes in seed development. Regulation of *AGL15* and *STK* expression seems unaffected by *ABS*, *SHP1* and *SHP2* loss of function. However, as in the bud stage, *FUL* is up-regulated in *tt16* mutants suggesting that the negative regulation of *FUL* by *ABS* remains unchanged by fertilization.

Our expression analysis shows that five MADS-box genes known to play important roles in ovule and seed development in part interact genetically in a complex network that changes components and direction of regulation in a stage-dependent manner (Fig 6C). While *ABS* directly or indirectly represses *SHP1* or *SHP2* expression, *ABS* is repressed by *SHP1* and/or *SHP2* only before fertilization. *FUL* repression by *ABS* was observed in both stages. *AGL15* and *STK* expression does not seem to be significantly affected by the loss of ABS and/or SHP1 and SHP2 in floral buds or siliques.

## Discussion

Our work has provided quantitative data on the phenotypic effects of *ABS* loss of function in combination with loss of *SHP1* and *SHP2*. While ovule development commences in *tt16* mutants almost as efficiently as in the wild type, with the exception of a single integument layer sometimes not properly formed, we see deleterious effects of the *tt16* mutation after fertilization in inner seed coat layers and endosperm development. The *SHP1* and *SHP2* genes on the other hand are required for proper timing and synchronization of ovule development and/or the fertilization process which occurs more erratic in *shp1 shp2* mutants. Additionally, this work shows that *SHP1* and *SHP2* play an important role in seed coat differentiation.

### *ABS* is involved in the interplay between endosperm and seed coat development

Ovule development ends after megagametogenesis with the generation of the third inner integument cell layer by periclinal divisions of the innermost ii1 layer. The work presented in Fig 4 shows that *ABS* is essential for this process as in the *tt16* mutants, the ii1 layer divides in an uncoordinated way resulting in a patchy distribution or locally missing ii1’ layer. Several details of our work indicate that the formative divisions of the inner integument are controlled by strictly unipolar positional signaling: (i) the successive divisions giving rise to the ii1´ layer always proceed from the chalazal to the micropylar end (Fig 4, S2 Fig; [46]), (ii) after fertilization, formative divisions continue only at the micropylar side of the developing seed (S4A–S4J Fig), (iii) failure of formative divisions at the chalazal end of the *tt16* mutant ovules (Fig 4E) cannot be compensated for during seed development so that the ii1´ layer is lacking on the chalazal side but reaches further down towards the micropylar end (Fig 3A and 3B), and (iv) gaps in the ii1´ layer caused by the patchy layer initiation in the *tt16* mutant ovules (Fig 4F) can be filled during further development, as the irregular ii1´ layer phenotype was no longer observed with developing *tt16* mutant seeds. However, while *ABS* controls the spatial position of formative divisions, it normally does not affect their number.

While ovule development and fertilization are largely unaffected by the loss of *ABS* function, around half of the *tt16* seeds show defects after fertilization. In these seeds, and also in the triple mutant, peripheral endosperm nuclei are not formed (Fig 2F and 2J) or only few endosperm nuclei are produced (Fig 2G and 2K) resulting in developing seeds that fail to expand (Fig 1E and 1G) and that will ultimately deliver less nutrients to the developing embryo resulting in seedlings that suffer from small size, fused organs, and lack of organs (Fig 1N–1R). But remarkably, the seedling defects were found in particular with the *tt16 shp1 shp2* triple mutant and not with the *tt16* single mutant (Fig 1B), although endosperm defects occurred in both plant lines.

*ABS* expression was observed in the inner integument and the nucellus at the chalazal end (Fig 5A), and also *SHP2* expression can be observed in ovules, but seems more broadly expressed [50]. But while *SHP1* and *SHP2* mRNA abundance remains at high levels in the seed coat and endosperm throughout seed development [54], *ABS* mRNA can hardly be detected after fertilization and can be seen only in the chalazal endosperm and no expression was found in the embryo (Fig 5C and 5D; [55, 54]).

Other important regulators of endosperm size and inner seed coat development are the *HAIKU* (*IKU*) genes [10]. Loss of *IKU* gene function also results in dramatically decreased endosperm size with seeds that are smaller but are shaped similarly to wild type seeds. As expected, the *IKU* genes are expressed after fertilization [10], while *ABS* expression decreases dramatically after fertilization (Fig 5C and 5D). Another important difference in phenotypes between the two mutations is that the inner seed coat layers of *tt16* are longer and thinner than in the wild type (Fig 3C–3J, S3F, S3G and S4K–S4V Figs) while in the *iku* mutants they are shorter [10]. This suggests that a reduction of the amount of endosperm in the developing seed may not necessarily coincide with cells of the inner seed coat layers being shorter, but also with elongated, thinner cells with lower cytoplasm density. The results presented in this work on bleached seeds show that the ii1 cells of *tt16* and *tt16 shp1 shp2* mutants show a less dense cytoplasm. This is in accordance to previous work suggesting that the ii1 cells of the *tt16* single and triple mutants may have the identity of parenchyma rather than endothelium cells and that this may be a more crucial character for endosperm development than cell shape (Fig 3C–3J, S4K–S4V Fig, [27, 28]). Altered cell identity may cause altered gene expression and, consequently, different influences on endosperm and embryo development. This was also shown previously for the differential regulation of the *BANYULS* promoter in the different regions of the curving zone which also coincides with different cell division behavior (called region 2/div+ and region 2/div- by Debeaujon et al., 2003 [28]).

However, we cannot discriminate between the two possible scenarios: (i) that *ABS* directs both, endosperm proliferation and inner seed coat development. One hint towards this hypothesis comes from the expression analysis presented in Fig 5C and 5D and Xu et al., 2016 [48] where the ABS protein can be detected in the chalazal endosperm after fertilization but not in the seed coat layers. While *ABS* is expressed in the chalazal part of the endosperm, it directs mainly the development of the peripheral part of the endosperm, at least at the chalazal end of the seed. Or (ii), the modified distribution of inner integument cell layers and the altered ii1 cell identity caused by *tt16* indirectly result in fewer endosperm nuclei and later in fewer endosperm cells. One may then hypothesize that *ABS* directs endosperm development indirectly by ensuring proper identity of the inner integument layers which, in turn is required for endosperm formation. Thus, defects in seed coat proliferation and morphology before fertilization may cause defects in endosperm formation after fertilization [11], and *ABS* is involved in the regulation of this process. This hypothesis is also supported by recent work from Xu et al., 2016 [48] who show that nucellus degeneration after fertilization is required for chalazal endosperm development and positioning and that *ABS* plays a major role in this process.

### Seed coat differentiation requires *ABS* and *SHP1/SHP2*

Seed coat development is essentially a consequence of fertilization. Unlike the embryo, sexually formed endosperm triggers the differentiation of integuments into endothelial cells. This has been shown in *kokopelli* mutant where only a single sperm cell is produced by the male gametophyte leading to a single fertilization event. Here only when the central cell is fertilized the seed coat development commences but not when the egg cell is fertilized [56]. Furthermore, Roszak et al. (2011) [57] showed that polycomb group proteins that are expressed in the ovule integument suppress seed coat formation in the absence of fertilization and the authors identified *AGL62* as the gene responsible in the formation of such mobile signal that establishes the communication between endosperm and integument. This indicates that there is a cross-talk between the fertile part and the maternal sporophytic tissue. Our results also show the association between defect in integument development and endosperm proliferation in *tt16* mutant background genotype. Since *ABS* transcripts are present in the ovule integument but not in the seed coat, we speculate that signaling molecules originating from the integuments spread to the female gametophyte which are essential for the peripheral endosperm proliferation. As the ABS protein is unable to move between endothelium cells it has been suggested earlier to initiate a signaling cascade spreading across nucellus and endothelium [48]. A similar signaling pathway can be proposed to remain until later in development allowing peripheral endosperm proliferation.

One role of *ABS* in seed coat development has been described already by Nesi et al. (2002) [27] and Debeaujon et al., (2003) [28] as regulator of the important PA pathway gene *BANYULS* (*BAN*). And indeed, *tt16* seeds are straw colored due to the lack of PA except for a small brown dot at the chalazal part of the seed coat (Fig 1E, S1C Fig). In the *tt16* mutant, residual expression of *BAN* remains in the chalazal bulb [27] in a region where *ABS* is expressed exclusively after fertilization. This suggests an additional spatial component of the ABS-BAN interaction as the late expression domain of *ABS* seems to not influence *BAN* transcript abundance.

The PA’s accumulate in the endothelium where also the *tt16* phenotype is occurring in particular (Fig 3, [28, 27]). But *tt16* seeds are also impaired in mucilage production (Fig 1J and S1H Fig) suggesting that *ABS* also affects differentiation of the outer layer of the seed coat in the last phases of seed development without ever being expressed there. These observations suggest a transport mechanism from the inner seed coat layers to the outer ones or, alternatively, from the chalazal end to the outer seed coat layer(s) [58]. This transport mechanism is most likely not relevant for ABS, as the GUS staining in our experiment (Fig 5C and 5D) is not only dependent on the *ABS* promoter activities but also on the ABS protein stability and transport. And a translocation to the outer integuments or a specifically long persistence in the inner seed coat layers cannot be observed, moreover, late *ABS* expression was found in the chalazal endosperm rather than in the chalaza region itself (Fig 5C and 5D). More likely, an unknown target of ABS mediates the interaction with the outer seed coat layers and bridges the temporal gap between *ABS* expression and exertion of its function.

The *SHP* genes have so far not been reported to play a role in seed coat development. However, Fig 1K and S1I Fig show that mucilage production or release in *shp1 shp2* seeds is patchy and the mucilage layer is thinner than in wild type. A recent review by Francoz et al. (2015) [59] summarizes the genetic network known to date involved in the regulation of mucilage production and the morphological changes to the epidermis allowing mucilage production, storage, and release, however, this network does not incorporate any MADS-box transcription factors. In *tt16 shp1 shp2* mutants, hardly any mucilage can be observed (Fig 1L and S1J Fig) and these seeds cannot be moistened, rather, they float on water surfaces. Quantitative data for seed coat expression can be derived from Belmonte et al. (2013) [54] indicating a strong and persisting *SHP1* and *SHP2* expression in the seed coat and Khan et al. (2014) [60] place *SHP1* and *SHP2* into a regulatory network directing seed coat development based on co-expression analysis. Here, we provide experimental evidence that *SHP1* and *SHP2* indeed have an important role in the differentiation of the epidermal seed coat layer.

In summary, *ABS* is required for PA accumulation in the seed coat. *ABS* is also, together with *SHP1* and *SHP2*, involved in the regulation of mucilage production (Fig 1J–1L) which is important during seed germination as the seeds remain in contact with the soil when the mucilage has expanded by accumulating water. The physiological role for mucilage production remains unclear, as several functions have been proposed but little experimental evidence supports them. Mucilage maybe required for sinking the seed in water and for its adhesive properties leading to efficient long-distance transport by animals [61]. And thirdly, *ABS* is required for endosperm development that later supplies nutrients to the embryo and a sufficient amount of endosperm is thus a prerequisite for seedling development even well after germination (Fig 1N–1R).

In the natural environment of *A*. *thaliana* seeds, which is characterized by multiple stressors like osmotic challenges, water scarcity, UV-stress, flooding, and many more, *ABS* gene function is, together with *SHP1* and *SHP2*, most likely critical for the most vulnerable phase of the plant’s life—seed germination and seedling survival.

### A MADS-box gene regulatory network for seed development

As most transcription factors, the proteins encoded by MADS-box genes bind to promoters of target genes, which in many cases are also transcription factors, but also genes involved in hormone response, cellular differentiation, pattern formation and many more [62]. The phenotype and expression analysis presented here provide an insight in the genetic interaction of selected ovule and seed morphogenesis regulators.

The genetic interactions of MADS transcription factors in seed development are not well understood to date and only few genetic interactions have been described so far, such as the negative regulation of *SHP1* and *SHP2* by *FUL* during dehiscence zone development at very late stages of fruit maturation [45]. Recent analyses have shown by ChIP and microarray analyses that *ABS*, *SHP1*, *SHP2*, and *AGL15* are direct targets of the carpel identity gene *AGAMOUS* (*AG*), but *FUL* and *STK* are not [52]. However, *AG* is expressed comparatively early during flower development, at inception of the carpel meristem and remains expressed throughout carpel development. Specifically in the late stages of ovule development, *AG* expression is found in the inner cell layers of the inner integument [63]. During ovule development, *AG* and *ABS* are co-expressed indicating that the regulation of *ABS* by *AG* may persist during ovule development.

The expression analysis presented in this work (Fig 6) suggests that most of the genes analyzed in this work exert their function as repressors of the other transcription factors involved, as in the case of *ABS*—*SHP1/SHP2* and *ABS*–*FUL*, and these are in part of mutually repressive relationship. However, *SHP1*, *SHP2*, and *FUL* are also expressed in the valve margins and valves, respectively, but *ABS* expression is restricted to ovules. Thus, the genetic network involving *ABS* is restricted to ovule and seed development.

While the repression of *FUL* and *SHP1/SHP2* expression by *ABS* occurs before and after fertilization, the action of *SHP1* and/or *SHP2* changes to some extent after fertilization. This change of regulation may not come as a surprise as the developmental program changes upon fertilization and *SHP1* and *SHP2* are apparently part of this remodeling process.

## Conclusions

Our work suggests that *ABS* is not only required for the regulation of PA accumulation in the seed coat but that its functions are of much broader impact: they range from positively regulating coordinated cell divisions in the inner integument layer of the ovule, nuclear endosperm proliferation and thus seed cavity expansion, to regulating endothelium cell morphology, and to its influence on mucilage release of the seed coat epidermal cell layer. Novel roles could also be identified for *SHP1* and *SHP2*: these genes are required for synchronized ovule maturation and subsequent fertilization/seed maturation, seed mucilage production, and have an inhibitory effect which is antagonistic to the *ABS* function in controlling formative divisions and cell shape of the inner integument and seed coat layers. Unlike *SHP1* and *SHP2*, which are the result of a rather recent genome duplication and specific to the Brassicaceae [64], orthologs of *ABS* were present in the most recent common ancestor of gymnosperms and angiosperms [25]. The newly discovered function of *ABS* in endosperm development and its coordination with inner seed coat formation provide a hint towards original function of B_sister_ genes, which may be conserved throughout the seed plants. The ability to protect the zygote by integuments from the environment and to produce a nutritive tissue embedding and supporting the embryo and providing nutrients to the seedling after germination is one of the key traits to seed plant success to which the B_sister_ genes have contributed significantly.

## Materials and Methods

### Plant material and growth conditions

Col-0 and Ws-4 wild type plants, the *tt16-1* T-DNA insertion line [27] obtained from the INRA-Versaille mutant populations whose parental line is Ws-4 (see information of the Nottingham Arabidopsis stock centre), and the *shp1-1 shp2-1* double mutant T-DNA insertion line in the Col-0 background [36] were obtained from the Nottingham Arabidopsis stock centre (NASC) and were genotyped by PCR, the allele specific oligonucleotides are listed in S1 Table.

A promoter GUS line for *ABS* (*pABS*::*ABS*:*GUS*) was created by cloning 3.5 kb *ABS* promoter region (entire intergenic region between the 5’ gene and *ABS* start codon) and 1.8 kb *ABS* genomic locus in frame with *GUS* in the pGWB633 plasmid [65] using the Gateway^®^ recombination system (Life technologies GmbH, Darmstadt, Germany). *A*. *thaliana* Col-0 plants were transformed by *Agrobacterium tumefaciens* assisted floral dipping as mentioned previously [66] with the addition of 100 μM acetosyringone and 25 g l^-1^ of sucrose to the dipping medium. F1 transformants were selected on soil by spraying 1 week old seedlings with 300 μM Basta^®^ (Bayer GmbH, Germany) at an interval of 3 days for 3–5 times.

All plants were sown on standard potting soil with perlite (ED73, H. Nitsch & Sohn GmbH & Co.KG, Kreuztal-Eichen, Germany), stratified for three days at 4°C in the dark and then moved to growth cabinets set to long day conditions (16 h light: 150 μmol m^-2^ s^-1^ photons, 22°C; 8 h dark, 18°C; relative humidity ~60%).

For cotyledon greening assay on soil, 300 seeds per line were stratified and sown on soil as described above. Seedlings were scored as viable when the cotyledons were fully expanded and the first set of true leaves appeared. The average number of germinated seedlings and the standard deviation was calculated and the statistical significance of the difference between the wild type and mutant seed germination rates was analysed with a single factor ANOVA.

### Ovule and seed morphology analyses

Whole mount analyses of cleared ovules and seeds were performed to visualize the developing integuments and embryo sacs, as well as seed coats, endosperm tissues and embryos. Stage 12 flower buds (stages defined after Smyth et al., 1990 [67]) were randomly collected from 5 plants per genotype and stage 16 to 17A siliques were selected from at least 3 plants per genotype. For conventional light microscopy with differential interference contrast (DIC) optics, tissue fixation in 10% glacial acetic acid and 90% ethanol, dehydration and clearing with chloral hydrate was carried out as described in Yadegari et al. (1994) [68]. The substructures of dissected ovules and developing seeds were carefully analyzed with a Leica DM55003 microscope and photographed in different focus planes using the above mentioned camera system. In order to demonstrate all the details observed, most figures were mounted from DIC micrographs of different focus planes using Corel PHOTO-PAINT V10.0 (Corel Corporation, Ottawa, Canada). Ruthenium Red staining was performed according to Dean et al. (2011) [41] without shaking of the seeds.

For the observation of wild type and mutant ovules with a confocal laser scanning microscope (CLSM), stage 12 flower buds were either quickly fixed and dehydrated following the protocol of Yadegari et al. (1994) [68], or they were slowly fixed for 26h in a solution of 4% glutaraldehyde in 12.5 mM cacodylate, pH 7.2, and dehydrated in a graded ethanol series with 20 min per step (modified after Shi et al., 2005 [69]). All buds were stored at 4°C and after a gradual rehydration, they were stained for five days in 5 mg/ml propidium iodide dissolved in water, before they were dehydrated again (see [70]). Pistils were isolated from the quickly fixed flowers and dissected ovules were cleared on a microscopic slide with chloral hydrate. The slowly fixed flowers were cleared in 2:1 (v/v) benzyl benzoate:benzyl alcohol for several hours before ovules were dissected and mounted with immersion oil on a microscopic slide [69]. All propidium-iodide stained ovules were observed with a 63x oil immersion objective at a Leica TCS SP5 VIS CLSM (Leica Microsystems, Mannheim, Germany) using excitation with the 488nm line of the argon laser and an emission window from 570 to 610 nm. Leica Application Suite software was used for automated z-stacks and image processing.

### Expression analysis

GUS staining for the *ABS* promoter GUS line was performed as described previously [71] with clearing as described in Yadegari et al. (1994) [68] and documented by the microscope and camera mentioned above.

For qRT-PCR, total RNA was isolated from *Arabidopsis thaliana* floral buds (mixture of stages 1–12) and stage 17A siliques using the plant-rna-OLS^®^ Kit (Omni Life Science, Germany). 1 μg of RNA and random primer were used to synthesized first strand cDNA following the protocol of RevertAid^™^HMinus First Strand cDNA Synthesis Kit (Fermentas, Germany). qRT-PCR was performed using Roche LightCycler^®^ 480 system (Roche, Germany) with a total reaction volume of 20 μl containing 5 μl of 1:50 diluted cDNA template, 1 μl of each PCR primer (10 μM), 3 μl double distilled sterile water and 10 μl SYBR Green I Master (Roche) and assayed with following cycling condition: initial heating of 95°C for 5 min, 45 cycles of 10 s at 95°C for denaturation, 10 s at 60°C for primer annealing, and 10 s at 72°C for extension. Exon-intron spanning primers were subjected to primer efficiency tests and are listed in S1 Table. *ACTIN* and *EF1-α* were used as reference genes for floral bud and stage 17A tissue. Three biological replicates with two technical replicates were carried out for each data point and analysis of data was performed following the ΔΔCT (cycle threshold) method [72].

### Lipid analysis

Seed fatty acid composition was analyzed on 0.1 g pooled seed samples from each individual genotype. Fatty acid methyl esters (FAME) were obtained from seed oil after petroleum ether extraction and transesterification with sodium methylate. FAME were transferred in iso-octane and analyzed in a capillary gas chromatograph (TRACE GC, Thermo Finnigan, Italia) equipped with an autosampler (AS 3000, Thermo Scientific) and configured as follows: Capillary column BPX70 (0.53mm ID, 30m length, SGE Analytical Science, Australia), 0.8μl injection volume, injector temperature 260°C, FID temperature 280°C, gradient oven temperature 160°C to 220°C in 12 min, helium flow rate 3.1 ml min^-1^. Individual fatty acids were identified and quantified using an internal FAME standard (Rotichrom ME 51; Carl Roth, Germany) and recorded as the percentage of the total fatty acids per sample. To test for statistical significance of the differences in the observed seed fatty acid composition an ANOVA together with a LSD-test were performed using IBM SPSS statistics Version 21 (IBM Software, Armonk, NY, USA).

## Supporting Information

S1 Table List of oligonucleotides used in this study. (PDF)

S2 Table P-values documenting the statistical analysis (Post-Hoc-tests) of the lipid analyses in pairwise comparisons of the plant lines (Col-0, Ws-4, *tt16*, *shp1 shp2*, *and tt16 shp1 shp2)*. Yellow: p<0.05; blue: p<0.001; green: p<0.0001.
(PDF)

S1 Fig Seed morphology shown enlarged for Ws-4 (A), Col-0 (B), *tt16* (C), *shp1 shp2* (D), *and tt16 shp1 shp2* (E) seeds. Seed coat mucilage release illustrated by Ruthenium Red staining showing an enlarged portion of the seed coat. Ws-4 (F); Col-0 (G); *tt16* (H); *shp1 shp2* (I); *tt16 shp1 shp2* (J). Scale bars in (A)-(E): 500 μm; (F)-(J): 200 μm.
(EPS)

S2 Fig Normal ovule and seed development of Ws-4 and Col-0 wild type plants largely corresponds to that of the Ler-0 accession which has been described in detail by Schneitz et al. (1995) [44]. The figures show developmental stages with terminology following Beeckman et al. (2000) [46]. (A), very young Ws-4 ovule, abbreviations: outer integument (oi), nucellus (n), outgrowing inner integument (ii). (B), young Ws-4 ovule during early megagametogenesis indicating the chalazal end (ch) and the micropyle (mi). The site where the first two periclinal divisions of the ii1 layer had occurred at the chalazal end at the abaxial side of the ovule (between arrowheads) is shown enlarged in the insert. (C), immature Col-0 ovule with a developing embryo sac (e). Arrowheads mark the start and the end of the formative divisions which gave rise to the additional ii1´ layer at the abaxial side and the adaxial side of the ovule, respectively. (D), Ws-4 ovule with an almost mature embryo sac showing three antipodal cells (black circles) and two polar nuclei (white circles). The egg cell and its synergids are located at the micropylar end. Arrowheads mark the start and the end of the ii1´ layer at the abaxial side and the adaxial side of the ovule. The curving zone at the abaxial side is marked with a double headed arrow. (E), Col-0 seed with a globular embryo and properly developed endosperm consisting of micropylar endosperm (black arrowhead), chalazal endosperm (CE), and the syncytial peripheral endosperm which contains many nuclei (nPE). The seed coat originates from the ovule integument layers in the curving zone (oi2, oi1 and ii2, ii1´, i1). The innermost layer ii1 has developed into the endothelium whose cells have a thick and almost isodiametric shape and possess a dense cytoplasm (white arrowhead). (F), Col-0 seed with a heart stage embryo and properly developed endosperm consisting of micropylar endosperm (arrowhead), chalazal endosperm (CE), and peripheral endosperm which starts to cellularize at this developmental stage (cPE). (G), Ws-4 seed with an embryo at heart to torpedo stage which is surrounded by cellular peripheral endosperm (cPE). Note the reduced density of the cytoplasm of endothelium cells (ii1 layer, arrowhead). (H), Ws-4 seed with a torpedo stage embryo. The peripheral endosperm degenerates except for the outermost layer(s) attached to the seed coat, which can be considered as aleurone layer(s) (AL). CE marks the chalazal endosperm. The shape of the endothelium cells (ii1 layer, white arrowhead) has changed to thin and elongated. Scale bars are 50 μm.
(EPS)

S3 Fig Seed anatomy of Ws-4 and Col-0 wild type plants and of *tt16*, *shp1 shp2*, and *tt16 shp1 shp2* mutants. (A), Properly developed seed of a Col-0 plant with an early globular embryo. The syncytial peripheral endosperm contains many nuclei (e.g. circles). (B) to (E), unfertilized ovules/very young seeds and malformed seeds found in stage 16 and 17A siliques of different mutant lines besides properly developed seeds harboring octant, globular or early heart embryos. (B), rare case of unfertilized ovule/very young seed with a normal appearance in *tt16* siliques. (C), weak *tt16 shp1 shp2* seed phenotype containing an early globular embryo and properly developed micropylar endosperm (arrowhead) in a large seed cavity but only few peripheral endosperm nuclei. (D), seed of a *tt16 shp1 shp2* plant with well-developed micropylar endosperm (arrowhead) surrounding the heart stage embryo in a large seed cavity, but only one layer of peripheral endosperm cells attached to the seed coat (e.g. circles); the inlay (E) shows the inconspicuous chalazal endosperm in another focus plane (arrowhead). Scale bars (A)-(D) are 50 μm. (F) and (G), quantitative analysis characterizing the cell shape of the two innermost seed coat layers ii1 (endothelium) and ii1´ in properly developed seeds harboring globular or early heart embryos of stage 16 and 17A siliques. The same seeds were used for the data collection shown in Fig 3, but here only those seeds were considered for quantification, in which the ii1´ layer has at least been partially formed by periclinal divisions of the innermost layer ii1. Cell shape of the seed coat layers was investigated at the chalazal side of the seeds (F) and on the micropylar side (G). Classification as normal isodiametric cell shape or as thin, elongated cell morphology was performed according to the examples shown in Fig 3C–3J and S4K–S4V Fig.
(EPS)

S4 Fig (A)–(J), temporal analysis of the post fertilization development of the ii1´ seed coat layer of well-developed seeds of stage 16 and 17A siliques. Percentages of seeds with, without, and with only partially developed ii1´ layer, or with a locally occurring additional fourth layer derived from the inner integument on the chalazal side (A–E) and on the micropylar side (F–J). Developmental stages of the seeds are defined according to the embryo stages they contain (after [73]); stage 1: seeds with octant stage to early globular embryos, stage 2: seeds with mid globular embryos, stage 3: seeds with late globular and transition stage embryos, and stage 4: seeds with heart stage embryos. Tendencies in ii1´ layer development can be seen, although it is not strictly coupled to embryo development, and individual seeds with varying seed coat layer anatomy were found at all developmental stages in every plant line (see also K-P). Ws-4 accession (A); Col-0 accession (B); *tt16* (C); *shp1 shp2* (D); *tt16 shp1 shp2* (E); Ws-4 (F); Col-0 (G); *tt16* (H); *shp1 shp2* (I); *tt16 shp1 shp2* (J). (K)–(V), documentation of the seed coat layer anatomy at the micropylar side of well-developed seeds with globular to heart stage embryos. Seeds were collected from siliques at stage 16 and 17A. The DIC micrographs show Ws-4 seeds in which the ii1´ cell layer is almost completely developed (K), partially missing (L), or almost entirely missing (M) on the micropylar side, and Col-0 seeds in which the ii1´ layer is partially missing (N) or almost entirely missing (O, P). Arrowheads mark the respective sites where the ii1´ layer ends and the two innermost integument layers converge into one. (Q), anatomy on the micropylar side of the developing *shp1 shp2* seed coat layers resemble those of the Col-0 wild type and in most seeds the ii1´ layer is partially missing (below arrowhead). With the majority of *tt16* seeds, the ii1´ cell layer is completely developed on the micropylar side (R). In comparison to all other plant lines, partially missing ii1´ layers, which do not reach the micropylar end, were less frequently found (below arrowhead in S). The two inner *tt16* seed coat layers mostly have a thin and elongated shape. The lack of the dense cytoplasmic content in the innermost ii1 layer is another typical feature (R), which was also often found, if the *tt16* ii1 cells do not exhibit the characteristic thin and elongated shape (S). Seed coat anatomy on the micropylar side of the *tt16 shp1 shp2 triple* mutant might be regarded as a phenotypic intermediate of the Ws-4 wild type and the *tt16* mutant. The ii1´ cell layer is completely developed (T), partially missing (below arrowhead in U), or entirely missing (below arrowhead in V). With almost identical frequencies as observed for the Ws-4 seeds (M, see S3G Fig), the ii1´and ii1 cell layers are composed of cells with a thin and elongated shape (T-V). In contrast to the typical wild type seeds, the ii1 layer of triple mutant seeds usually has no dense cytoplasm. Scale bars in (K-V) are 50 μm.
(EPS)

S5 Fig Irregular embryo development seldom observed in seeds of stage 16 and 17A siliques harboring globular embryos. (A), Normally developed Col-0 wild type embryo with a long suspensor and regularly sized protoderm cells. (B) and (C), Ws-4 wild type embryo and *shp1 shp2* mutant embryo, respectively. Black arrowheads point at individual protoderm cells which are swollen and cause a slightly irregular shape of the embryo outline. (D) to (F), *tt16* mutant embryos; (G) to (I), *tt16 shp1 shp2* triple mutant embryos. Black arrowheads mark protoderm cells which are swollen to varying degrees. Conspicuously short suspensors (white arrowheads) represent another defect which may hinder the unrestrained embryo development in the seed cavity. Jamming the embryo at the micropylar end (E and H) may cause mechanical problems (F) leading to early embryo abortion (I). However, the very low number of seeds in which the suspensor defect was observed would by far not explain the large proportion of seeds with developmental defects found in the *tt16* mutant and in the *tt16 shp1 shp2* triple mutant (cf. Fig 2A). Endosperm defects seem to be more common (cf. Fig 2G, 2J and 2K). Scale bars are 50 μm.
(PDF)
