# Supplementary material for: The MADS Box Genes ABS, SHP1, and SHP2 Are Essential for the Coordination of Cell Divisions in Ovule and Seed Coat Development and for Endosperm Formation in Arabidopsis thaliana
Source: PLoS One. 2016 Oct 24;11(10):e0165075. doi: 10.1371/journal.pone.0165075 (PMC5077141; doi:10.1371/journal.pone.0165075)
Supplement: S1 Table — (PDF) [file pone.0165075.s001.pdf]

Supplemental Table 1: List of oligonucleotides used in this study

Name	Sequence (5'-3')	Purpose
abs_Fw	GGATTTTCATTTGGCCCAGAAGTT	Genotyping
abs_Rev	GTCGCAATCCGTTGGTATGC	Genotyping
abs_TDNA	CAACCCTCAACTGGAAACGGGCCGGA	Genotyping
shp1-1_Fw	GATGCACTCGAAATCAGCCAATTTTAG AC	Genotyping
shp1-1_Rev	GTGACGGAAGGAGGGTTGACG	Genotyping
shp1-1_TDNA	GTCTACTGATGAGTTGTCACTAGG	Genotyping
shp2-1_Fw	GAGGATAGAGAACACTACGAATCGTC	Genotyping
shp2-1_Rev	CAGGTCAAGTCAATAGATTCCCTAC	Genotyping
LB1.3	ATTTTGCCGATTTCGGAAC	Genotyping
RTq-AtABS_Fw	CTCTGTGACGCTCACATCGG	RTq-PCR
RTq-AtABS_Rev	AATGAGTTGAGGCATCCTGTTCTG	RTq-PCR
RTq-AtSHP1-Fw	AAG AAT GAG CTG TTA GTG GCA GAG	RTq-PCR
RTq-AtSHP1-Rev	CTTCGGCTATCTTTGCTCGCAG	RTq-PCR
RTq-AtSHP2-Fw	GCCAACAACAGTGTGAGAGGA	RTq-PCR
RTq-AtSHP2-Rev	AGTATTAGCTTCGGTGATGGTCGG	RTq-PCR
RTq-AGL11-Fw	CCAACAGGAATCTGATGGGAGAC	RTq-PCR
RTq-AGL11-Rev	GCAACTCATGCTTCTTGGACCT	RTq-PCR
RTq-AGL15_Fw	TCAGAGAGCGAAAGGAACGATTG	RTq-PCR
RTq-AGL15_Rev	TCTTGAACCTGTCTACGCAAGGT	RTq-PCR
RTq-AtFUL_Fw	GTCTGGTTTGCTCAAGAAAGCTC	RTq-PCR
RTq-AtFUL_Fw	CTCTCCATGCAAGAGTCGGT	RTq-PCR
RTq-At-Actin_Fw	AGTGGTCGTACAACCGGTATTGT	RTq-PCR
RTq-At-Actin_Re	GATGGCATGGAGGAAGAGAGAAAC	RTq-PCR
EF1_Fwd	TGAGCACGCTCTTCTTGCTTTCA	RTq-PCR
EF1_Rev	GGTGGTGGCATCCATCTTGTTAC	RTq-PCR
ABS_F_Gateway	GGGGACAAGTTTGTACAAAAAAGCAGG CTACTAACAATTAACCCCAAAAAATGAC	Promoter:GUS construct
ABS_R_Gateway	GGGGACCACTTTGTACAAGAAAGCTG GGTCATCATTCTGGGCCGTTGGATCGT T	Promoter:GUS construct
