# Supplementary material for: The MADS Box Genes ABS, SHP1, and SHP2 Are Essential for the Coordination of Cell Divisions in Ovule and Seed Coat Development and for Endosperm Formation in Arabidopsis thaliana
Source: PLoS One. 2016 Oct 24;11(10):e0165075. doi: 10.1371/journal.pone.0165075 (PMC5077141; doi:10.1371/journal.pone.0165075)
Supplement: S2 Table — Yellow: p<0.05; blue: p<0.001; green: p<0.0001. (PDF) [file pone.0165075.s002.pdf]

Table 2: P-values documenting the statistical analysis (Post-Hoc-tests) of the lipid analyses in pairwise comparisons of the plant lines (Col-0, Ws-4, *tt16*, *shp1 shp2*, and *tt16 shp1 shp2*). Yellow: p<0.05; blue: p<0.001; green: p<0.0001.

Palmitic acid

	Col-0	Ws-4	*tt16*	*shp1 shp2*
Col-0				
Ws-4	0,0316729			
tt16	0,0020700	0,1422053		
shp1 shp2	0,0001653	0,0000155	0,0000025	
tt16 shp1 shp2	0,0165171	0,6835744	0,2776935	0,0000111

Palmitoleic acid

	Col-0	Ws-4	*tt16*	*shp1 shp2*
Col-0				
Ws-4	0,4850209			
tt16	0,5617229	0,8490530		
shp1 shp2	0,0000001	0,0000001	0,0000001	
tt16 shp1 shp2	0,6311315	0,2532203	0,2825426	0,0000002

Stearic acid

	Col-0	Ws-4	*tt16*	*shp1 shp2*
Col-0				
Ws-4	0,1590527			
tt16	0,0001208	0,0000252		
shp1 shp2	0,6803093	0,0830022	0,0001958	
tt16 shp1 shp2	0,0002537	0,0000537	0,9104576	0,0004084

Oleic acid

	Col-0	Ws-4	*tt16*	*shp1 shp2*
Col-0				
Ws-4	0,0025432			
tt16	0,0000545	0,0000016		
shp1 shp2	0,0003482	0,1453626	0,0000006	
tt16 shp1 shp2	0,8967866	0,0030506	0,0000476	0,0004051

alpha-Linolenic acid

	Col-0	Ws-4	*tt16*	*shp1 shp2*
Col-0				
Ws-4	0,0001504			
tt16	0,0000000	0,0000000		
shp1 shp2	0,0397681	0,0027543	0,0000000	
tt16 shp1 shp2	0,0000001	0,0000055	0,0000001	0,0000004

Arachidic acid

	Col-0	Ws-4	*tt16*	*shp1 shp2*
Col-0				
Ws-4	1,0000000			
tt16	0,0000000	0,0000000		
shp1 shp2	1,0000000	1,0000000	0,0000000	
tt16 shp1 shp2	0,0000000	0,0000000	0,0009806	0,0000000

11-Eicosenoic acid

	Col-0	Ws-4	*tt16*	*shp1 shp2*
Col-0				
Ws-4	0,0002914			
tt16	0,9412156	0,0001695		
shp1 shp2	0,0000036	0,0000001	0,0000017	
tt16 shp1 shp2	0,0017398	0,0000051	0,0009043	0,0001670

unidentified FA

	Col-0	Ws-4	*tt16*	*shp1 shp2*
Col-0				
Ws-4	1,0000000			
tt16	0,0000000	0,0000000		
shp1 shp2	1,0000000	1,0000000	0,0000000	
tt16 shp1 shp2	0,0000000	0,0000000	0,0409354	0,0000000
