# Supplementary material for: The MADS Box Genes ABS, SHP1, and SHP2 Are Essential for the Coordination of Cell Divisions in Ovule and Seed Coat Development and for Endosperm Formation in Arabidopsis thaliana
Source: PLoS One. 2016 Oct 24;11(10):e0165075. doi: 10.1371/journal.pone.0165075 (PMC5077141; doi:10.1371/journal.pone.0165075)
Supplement: S5 Fig — (A), Normally developed Col-0 wild type embryo with a long suspensor and regularly sized protoderm cells. (B) and (C), Ws-4 wild type embryo and shp1 shp2 mutant embryo, respectively. Black arrowheads point at individual protoderm cells which are swollen and cause a slightly irregular shape of the embryo outline. (D) to (F), tt16 mutant embryos; (G) to (I), tt16 shp1 shp2 triple mutant embryos. Black arrowheads mark protoderm cells which are swollen to varying degrees. Conspicuously short suspensors (white arrowheads) represent another defect which may hinder the unrestrained embryo development in the seed cavity. Jamming the embryo at the micropylar end (E and H) may cause mechanical problems (F) leading to early embryo abortion (I). However, the very low number of seeds in which the suspensor defect was observed would by far not explain the large proportion of seeds with developmental defects found in the tt16 mutant and in the tt16 shp1 shp2 triple mutant (cf. Fig 2A). Endosperm defects seem to be more common (cf. Fig 2G, 2J and 2K). Scale bars are 50 μm. (PDF) [file pone.0165075.s007.pdf]

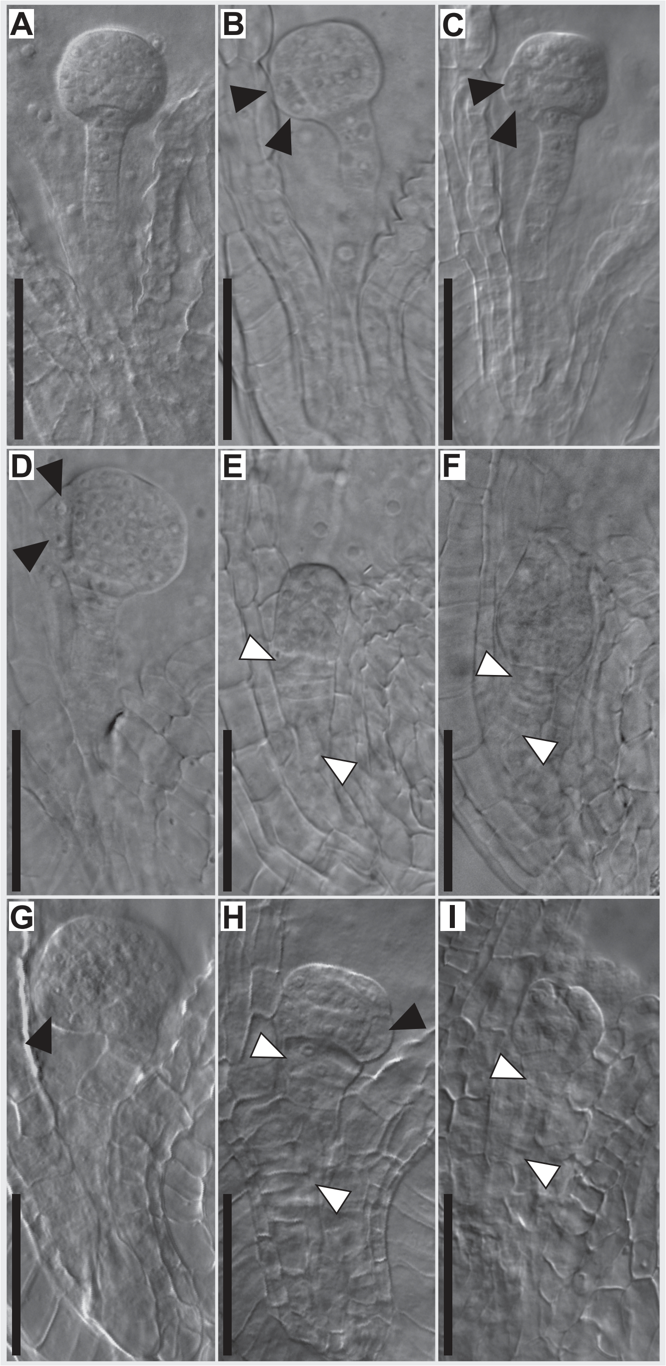

Supplemental Figure 5: Irregular embryo development seldom observed in seeds of stage 16 and 17A siliques harboring globular embryos. (A), Normally developed Col-0 wild type embryo with a long suspensor and regularly sized protoderm cells. (B) and (C), Ws-4 wild type embryo and *shp1 shp2* mutant embyro, respectively. Black arrowheads point at individual protoderm cells which are swollen and cause a slightly irregular shape of the embryo outline. (D) to (F), *tt16* mutant embryos; (G) to (I), *tt16 shp1 shp2* triple mutant embryos. Black arrowheads mark protoderm cells which are swollen to varying degrees. Conspicuously short suspensors (white arrowheads) represent another defect which may hinder the unrestrained embryo development in the seed cavity. Jamming the embryo at the micropylar end (E and H) may cause mechanical problems (F) leading to early embryo abortion (I). However, the very low number of seeds in which the suspensor defect was observed would by far not explain the large proportion of seeds with developmental defects found in the *tt16* mutant and in the *tt16 shp1 shp2* triple mutant (cf. Fig. 2A). Endosperm defects seem to be more common (cf. Figs. 2G, 2J, 2K). Scale bars are 50 µm.
